# The association of vimentin and fibronectin gene expression with epithelial-mesenchymal transition and tumor malignancy in colorectal carcinoma

**DOI:** 10.17179/excli2017-481

**Published:** 2017-07-10

**Authors:** Zohreh Niknami, Ali Eslamifar, Amirnader Emamirazavi, Alireza Ebrahimi, Reza Shirkoohi

**Affiliations:** 1 Department of Genetics, Faculty of Science, Islamic Azad University, Damghan, Iran; 2 Department of Clinical Research, Pasteur Institute of Iran, Tehran, Iran; 3 Iran National Tumor Bank, Cancer Institute of Iran, Tehran University of Medical Sciences, Tehran, Iran; 4 Department of Hematology, Faculty of Medical Sciences, Tarbiat Modares University, Tehran, Iran; 5 Cancer Biology Research Center, Cancer Institute of Iran, Tehran University of Medical Sciences, Tehran, Iran

**Keywords:** colorectal cancer, fibronectin, vimentin, qReal-Time PCR

## Abstract

Colorectal cancer is the most common malignancy of the gastrointestinal tract with very high mortality. One of the most distinguishing features for the establishment of an epithelial-mesenchymal transition phenotype is the alteration of mesenchymal markers expression and structural adhesion proteins. We evaluated the significance of vimentin and fibronectin gene expression in relation to invasion and metastasis in CRC patients. Tissue specimens were collected consecutively from forty-five colorectal carcinoma patients during surgeries. Tissues were divided into two separate parts for pathological and molecular assays. In order to histological staging, tissue sections were prepared from formalin-fixed paraffin-embedded blocks and stained with Hematoxylin and Eosin. To quantify gene expression, specimens were dissected and homogenized. Moreover, SW480, SW48, SW948, Caco-2, HT-29 and LS174T as human colon cancer cell lines were obtained and cultured, then molecular analyzing was performed. As results the expression of *VIM* gene increased in SW480, SW48 and SW948 while it decreased in Caco-2, HT-29 and LS174T. Moreover, *FN* was up-regulated in Caco-2, HT-29 and SW948, while it was down-regulated in SW480, SW48 and LS174T. In tissues, vimentin and fibronectin expression significantly increased in stromal cells, whereas vimentin decreased in colonic epithelial cells and fibronectin had no significant change. Vimentin and fibronectin expression were changed in tumor tissues. It was found an association between vimentin expression with age and tumor size; over-expression in older age and decreasing in larger tumor size. Furthermore, fibronectin over-expression is correlated to older age and high tumor stages; up-regulation with increasing age and high tumor stages.

## Introduction

It is well demonstrated that colorectal cancer (CRC) is one of the highly aggressive malignant tumors. CRC is the third cancer following lung and breast cancers in men and the second cancer in women worldwide (Armaghany et al., 2012[1]). It is also a cancer with very high mortality, the course of the disease results in death in about one of two CRC patients. Over 1.2 million new CRC cases, approximately 800,000 deaths are recorded annually (Jemal et al., 2011[6]; Sun et al., 2016[19]). Inherited familial CRC syndromes include Familial Adenomatous Polyposis (FAP) and Lynch Syndrome (Hereditary Non-Polyposis Colorectal Cancer [HNPCC]). The study of CRC syndromes has greatly assisted understanding of the molecular pathogenesis underlying sporadic CRC. Lynch syndrome (3-5 % of colorectal cancer), an autosomal dominant inherited disorder, is a disease in which pathogenesis is influenced by environmental exposures, genetic and epigenetic alterations (Sinicrope et al., 2016[18]). Some risk factors include lifestyle, dietary factors, side-effects of medical interventions, older age, family history and inherited genetic disorders (Lin, 2009[7]). Colorectal cancer ‎is genetically caused by accumulated mutations in multiple genes that regulate cell growth and differentiation. Mutations in DNA mismatch repair (MMR) genes lead to profound genetic instability including chromosomal instability (CIN) and microsatellite instability (MSI) (Buza et al., 2016[3]). Tumor development risk is increased through one of the most complex cellular events as multi-step cascading process involving infinite proliferation, invasion and immigration (Huang et al., 2016[5]). Recent investigations have focused on the epithelial-mesenchymal transition (EMT) in cancer invasion and metastasis (Toiyama et al., 2013[20]). These researches on the molecular genomic targets and biomarkers underpinning EMT regulation hold tremendous potential in identifying the subset of patients that are at highest risk of developing metastasis in CRC.

Several factors and genes are associated with the process of tumor angiogenesis, invasion, growth and metastasis in CRC. One of the most distinguishing features for the establishment of an EMT phenotype is up-regulated expression of mesenchymal markers and down-regulated expression of structural adhesion proteins (Micalizzi and Ford, 2009[9]). Vimentin gene (*VIM*) is a mesenchymal marker encodes 466 amino acids polypeptide (53 kDa), highly conserved protein belongs to type III intermediate filament family (Satelli and Li, 2011[16]). From studies and investigation, it is evident that vimentin is a multifunctional protein and its ability to interact with a large number of proteins makes it a potential regulator of several different physiological functions (Satelli and Li, 2011[16]). This protein is responsible for maintaining cell shape, integrity of the cytoplasm, stabilizing cytoskeletal interactions and involving in immune response (Ogrodnik et al., 2014[11]). It functions as an organizer of a number of critical proteins involved in attachment, migration, and cell signaling. Vimentin expresses during embryonic development and predominantly in the primitive streak stage, while its expression is limited to connective tissue mesenchymal cells, CNS and muscles in adult (Satelli and Li, 2011[16]). 

Fibronectin (FN) is an important extracellular matrix (ECM) glycoprotein (~440 kDa) with several alternatively spliced variants (Ou et al., 2013[12]). *FN* plays an important role in cell adhesion, migration and is involved in critical processes including embryogenesis, wound healing, blood coagulation, host defense and metastasis. *FN* has also been implicated in cancer growth and development. Therefore, this study was established due to our assumption that vimentin and fibronectin biomarkers may play roles in the CRC pathological process. To determine whether the gene expression of fibroblastic markers (*VIM* and *FN*) have changed in CRC tissue compared to normal colonic tissue, the level of mRNA for vimentin and fibronectin genes was measured. Consequently, we evaluated the significance of vimentin and fibronectin gene expression in relation to invasion and metastasis in CRC patients.

## Materials and Methods

### Cell lines and culture 

The human colon carcinoma cell lines SW480 (stage Duckes B, grade IV), SW48 (stage Duckes C, grade X), SW948 (stage Duckes C, grade III), Caco-2 derived from large intestine carcinoma (stage X, grade II), HT-29 (stage Duckes C, grade I) and LS174T (stage Duckes B, grade X) were obtained from the cellular bank department of Pasteur Institute (Tehran, Iran). The CRC cell lines were maintained and cultured in Dulbecco's modified Eagle's medium (Invitrogen Corp.), supplemented with 10 % fetal bovine serum (FBS) at 37 °C under 5 % CO_2_. The validity of cell lines was routinely monitored by analyzing a series of genetic and epigenetic specific markers for each cell line.

### Subjects and patients 

In this experimental study, sample size and power for patient and normal group were calculated forty-five and twenty specimens, respectively (95 % confidence level and 5 % confidence interval). Forty five fresh frozen samples (N=45) collected consecutively from Tumor bank of Iran Cancer Institute of Iran, Imam Khomeini Hospital Complex (Tehran, Iran). Informed written consent was obtained from patients and all procedures were performed according to the guidelines of scientific research ethical committee of Tehran University of medical science. 

### Histology and tissue collecting

Tissue specimens were divided into two separate parts for pathological and molecular assays. To quantify gene expression, specimens were dissected and stored immediately in -80 °C to inhibit ribonuclease enzyme and avoid RNA degradation.

In order to histological staging, tissue sections were prepared from formalin-fixed paraffin-embedded (FFPE) blocks and stained with Hematoxylin and Eosin (*H&E*). Finally, tissues were classified by anatomic pathologist team. 

### RNA isolation and cDNA synthesis

Tissue specimens (5 mg) were thawed and homogenized. Total RNA was extracted using High Pure RNA isolation kit (Roche, Germany). Last, the light absorbance at 260, 280 and 230 nm was measured using Nano-photometer 2000c (Thermo Science, USA). The RNA samples with optimum A260/A280 ratio and A260/A230 ratio equal or more than 1.7 were selected to synthesize complementary DNA.

Reverse transcription reaction was performed using RevertAid First Strand cDNA Synthesis Kit (Thermo Scientific, Lithuania). Reaction volume was 20 µL including; total RNA (1 μg≈11 µL), RevertAid RT 200 U/µL (1 μL), RiboLock RNase Inhibitor 20 U/µL (1 μL), Random Hexamer primer (1 μL), dNTP Mix 10 mM (2 μL) and Reaction Buffer 5x (4 μL). Then, samples were incubated for 10 min at 25 °C, 60 min at 42 °C and 5 min at 75 °C.

### Primer set design

Vimentin and fibronectin genes as fibroblastic markers were selected as target and Glyceraldehyde-3-phosphate dehydrogenase (*GAPDH*) gene was carefully chosen as internal reference gene. Genes sequence were attained, primers set were designed by *Primer3 *software. Finally, oligonucleotide sequences were compared and aligned with human whole genome and transcriptom to avoid secondary structure. Oligonucleotide sequences are shown in Table 1(Tab. 1).

### Quantitative Real-Time PCR analysis

Real-Time PCR assay based on free dye (SYBR Green-I) was carried out. All reactions were performed by Rotor-Gene Q apparatus (Qiagen, USA). Total volume for PCR reaction was 25 μl and including: 12.5 μl of SYBR Green-I PCR Master Mix 2x (TaKaRa, Japan), 1 μl of forward and reverse oligonucleotide (0.4 μM), 5 μl of cDNA template (60 ng/μl) and ddH_2_O (5.5 μl). Real-Time PCR programme was 30 sec at 95 °C (Taq enzyme activation), following by 5 sec at 95 °C and 20 sec at 60 °C for 40 repeats and melting curve analysis ramping from 65 °C to 95 °C and rinsing 1 °C per each detection.

Amplification efficiency for target and internal reference genes was validated using 2-fold dilution series of cDNA template as 400, 200, 100, 50, 25 ng. Standard curve was drawn by plotting the logarithmic input cDNA concentration versus mean Threshold Cycle (CT) and the slope was determined. PCR efficiency was calculated using formula; E %= [(10-(1/slope))-1] x 100. Expression level of target genes was calculated using comparative threshold cycle formula. The relative gene expression ratio (R) was calculated based on efficiency (E) and cross point (CP) of tumor tissues versus normal colon tissues using Pfaffl formula (Pfaffl, 2001[13]).


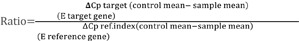


### Statistical analysis

The statistical procedures were included Mean ratio (M), Standard Deviation (SD), Confidence Intervals (95 % CI), Standard Error of Mean (SEM), Mann-Whitney assay and Kruskal-Wallis test. Mean values and associations between discrete variables were assessed using the ANOVA and the Chi-square (χ^2^) tests, respectively. The Kaplan-Meier method was used to estimate tumor recurrence or death from CRC. Significance level was set at *P* value <0.05. All mathematical analyses were performed using statistical package for the social sciences software (SPSS Inc. v. 22).

## Result

### Demographic data analysis

All CRC patients had no chemotherapy and were in stage I (n=12), stage II (n=18), stage III (n=7), stage IV (n=3) and stage X (n=5). Tumor tissue stages were categorized based on the Union for International Cancer Control (UICC) TNM classification system. The mean tumors' size was 4.70±2.53 cm. The primary site of samples were included cecum (n=10), rectum (n=16), Sigmoid Colon (n=8), Rectosigmoid (n=4), Ascending (n=3), Descending (n=3) and Transverse (n=1). The mean age of the patients was 55±15 years old (range 16-79), with 22 women and 23 men (Table 2(Tab. 2)).

### Real-Time RT-PCR validation

**Figure 1:**
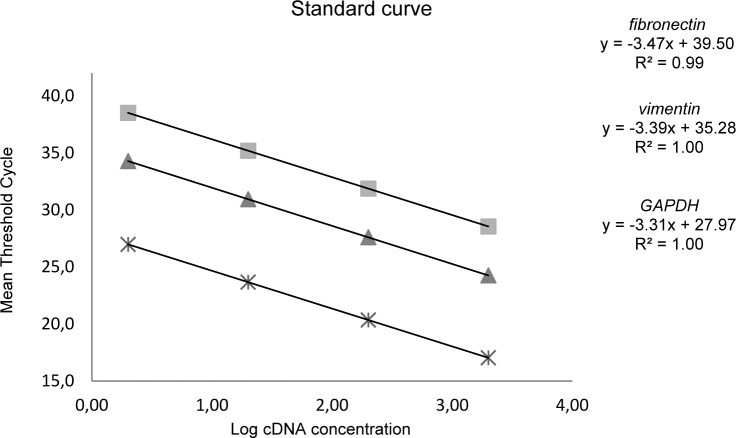
Standard curves for *VIM*, *FN* and *GAPDH*. Mean threshold cycle (X-axis) versus log cDNA concentration (Y-axis). Slopes, Y-intercept and determination coefficient for interest genes; vimentin (y= -3.39x + 35.28, R² = 1.00), fibronectin (y= -3.47x + 39.50, R² = 0.99) and GAPDH (y= -3.31x + 27.97, R² = 1.00).

**Figure 2:**
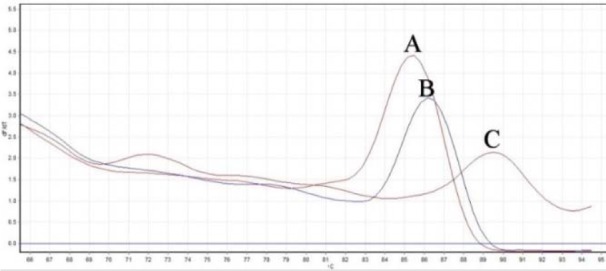
Melting curve analysis: *GAPDH*, *VIM* and *FN* were melted at 85.5 °C (A), 86.2 °C (B), 89.3 °C (C), respectively.

The slope of standard curves for *VIM*, *FN* and *GAPDH* genes were determined -3.39, -3.47 and -3.31, respectively (Figure 1(Fig. 1)). PCR efficiency (*E*) was calculated 97 % for *VIM*, 94 % for *FN* and 100 % for *GAPDH* gene. Melting curve analysis showed the specific amplicon for *VIM*, *FN* and *GAPDH* melted at 86.2 °C, 89.3 °C and 85.5 °C, respectively (Figure 2(Fig. 2)). The results of melting curves were confirmed by gel electrophorese analysis of PCR product.

### VIM and FN expression in CRC cell lines

**Figure 3:**
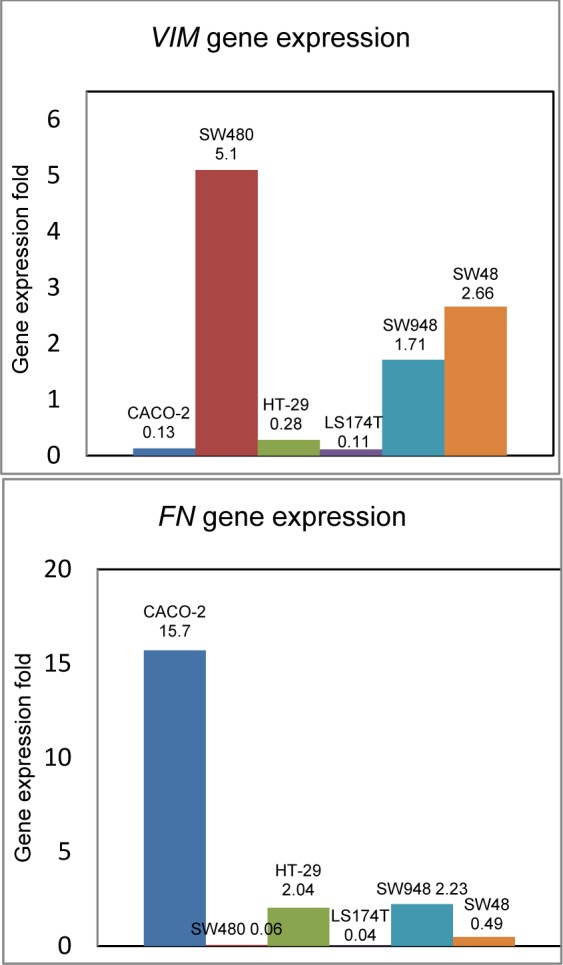
*VIM* and *FN* expression in CRC cell lines; based on pathology, morphology and cell behavior

Quantitative analysis showed that the expression of *VIM* gene increased in SW480 (5.10±1.37), SW48 (2.66±0.74), SW948 (1.71±0.29), while it decreased in Caco-2 (0.13±0.02), HT-29 (0.28±0.08) and LS174T (0.11±0.00) (Figure 3(Fig. 3)). 

*FN* exhibited different expression pattern compared to *VIM* expression. It was up-regulated in Caco-2 (15.7±1.33), HT-29 (2.04±0.41) and SW948 (2.23±0.36) cell lines, while it was down-regulated in SW480 (0.06±0.00), SW48 (0.49±0.09) and LS174T (0.04±0.00). 

**Figure 4:**
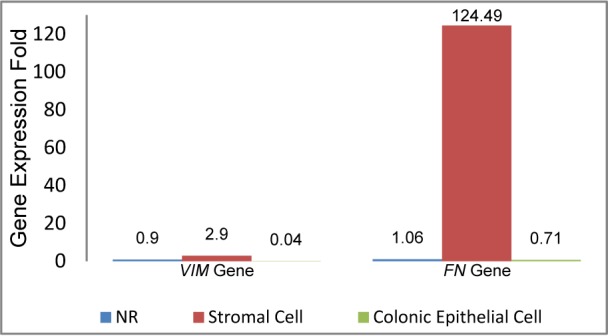
* VIM* and *FN* expression in CRC tissues; based on morphology, pathology, tumor behavior.

Data analysis showed that *VIM* had moderate gene expression profile in normal colonic tissue (mCT=25.84±1.01), while this represents a complex pattern in CRC tissues (Table 3(Tab. 3)). Quantitative Real-Time PCR revealed that *VIM* gene expression significantly increased about 3-fold (*P* value˂0.001) in stromal cells and decreased approximately 25-fold (*P* value˂0.001) in colonic epithelial cells of CRC tissues. *VIM* expression ratio for stromal cells and colonic epithelial cells was calculated 2.90±0.31 and 0.04±0.00, respectively (Figure 4(Fig. 4)). 

Fibronectin has low gene expression pattern in normal colonic tissue (mCT=32.54± 1.36). Quantitative results showed that *FN* gene expression increased approximately two logarithm fold in stromal cells, while had no significant change in colonic epithelial cells. *FN* expression ratio in stromal cells of CRC tissues was calculated 124.49±14.17 (*P* value˂0.001) and in colonic epithelium was 0.71±0.16 (*P* value˃0.05) (Figure 4(Fig. 4), Table 3(Tab. 3)).

## Conclusion

Colorectal cancer is the most common malignancy of the gastrointestinal tract that begins with transformation of normal epithelial cells to an adenoma, proceeding to *in situ* carcinoma, and eventually to invasive and metastatic tumor. Although, it is one of the well-studied cancers in recent years, however, the pathogenesis and molecular mechanism of CRC is not completely understood yet. It was thought that the key role of CRC involved in genetic alterations. Genetic and epigenetic mutations are most common factors in CRC and are the driving force of tumorgenesis (Armaghany et al., 2012[1]). Growing evidence suggests that some biomarkers play an important role in CRC development, progression and metastasis (Brenner and Rennert, 2005[2]; Ou et al., 2013[12]; Satelli and Li, 2011[16]; Toiyama et al., 2013[20]). Vimentin and fibronectin genes are responsible for maintaining cell shape, cytoplasm integrity, stabilizing cytoskeletal interactions, cell signaling, migration and cell adhesion (Micalizzi and Ford, 2009[9]; Ogrodnik et al., 2014[11]). These genes evaluated in various epithelial cancers including prostate cancer, gastrointestinal tumors, CNS tumors, breast cancer, malignant melanoma, lung cancer and other cancers (Satelli and Li, 2011[16]). Many studies and investigations have reported that in majority of the cancers, vimentin and fibronectin gene expression are altered and revealed an association between their expression and cancer aggressiveness (Mclnroy and Maatta, 2007[8]; Ngan et al., 2007[10]; Sethi et al., 2010[17]; Wei et al., 2008[21]; Williams et al., 2009[22]).

Essentially, expression of vimentin is mainly associated with metastatic phenotype and poor prognosis of the disease outcome. In the present study, fresh frozen CRC tissues were studied simultaneously with seven colorectal cell lines. The objective using cell line was the classification based on aggressiveness degree in cell lines and determination the expression profile of target genes in CRC cell lines compared to normal cell line. Our results showed that mRNA level of *VIM* increased in SW480, SW48 and SW948, while it decreased in Caco-2, HT-29 and LS174T. Fibronectin expression, moreover, had different pattern and behavior relative to *VIM* profile. It was up-regulated in Caco-2, HT-29 and SW948 cell lines, while it was down-regulated in SW480, SW48 and LS174T.

Quantitative data analysis showed that vimentin had complex pattern in CRC tissues. *VIM* expression is up-regulated in stromal cells and down-regulated in colonic epithelial cells of CRC tissues. It was found no statistical association between *VIM* gene expression with age, gender and tumor size (*p*˃0.05). However, the mRNA level of *VIM* gene was increased in low tumor grade (I-III) and was decreased (silenced or suppressed) in high tumor grade (III-X). Wei-Dong Chen and his colleagues using Immunohistochemical assay of vimentin expression in the human colon showed the absence of VIM protein in the colonic epithelial cells in both normal colonic crypts and in colon cancers. They showed vimentin gene is expressed in stromal cells within both normal and cancerous colon tissue (Chen et al., 2005[4]).

One of the most crucial steps in the tumor cell metastatic cascade is the acquisition of invasive capabilities, including destroying cell-cell junctions, degrading the cell matrix, and activating pathways that control the cytoskeletal dynamics of cancer cells. Fibronectin is an important Extracellular matrix (ECM) glycoprotein that plays an important role in cell adhesion, migration, cancer growth and development. Based on our finding, it is found that the *FN* gene expression is up-regulated in stromal cells of CRC tissue, while no significant change was observed in colonic epithelial cells. Data analysis revealed that fibronectin expression has no statistical differences with age, gender and tumor size (*p*˃0.05). But fibronectin mRNA level was dependent to tumor stages; up-regulation with low tumor stages (I-II) and decreased in high tumor stage (II-X). Recent studies on fibronectin expression reported that fibronectin expression levels are significantly higher in malignant tumors than benign tumors and normal tissues (Ou et al., 2013[12]; Rybak et al., 2007[14]; Saito et al., 2008[15]). Juanjuan Ou and his colleagues reported that fibronectin level was substantially higher in CRC compared to normal colon tissue. They also showed that this protein level was significantly higher in CRC tissues.

Many important features need to establish EMT phenotype. One of the most distinguishing features is alteration in expression of structural adhesion proteins (down-regulation) and mesenchymal markers such as vimentin and fibronectin (up-regulation). Though all findings indicate a future significance of vimentin and fibronectin genes for different malignancies with clinical relevance, however, more research will be necessary to particularly assess the major function of these genes in the process of EMT and tumorgenesis in colorectal carcinoma. 

## Notes

Ali Eslamifar and Reza Shirkoohi (Imam Khomeini Hospital Complexes - Cancer Institute - Cancer Biology Research Center, Tehran-Iran, Zip Code: 1419733141; Tel: 0098-21-66914545, Fax: 0098-21-66581638, E-mail: rshirkoohi@tums.ac.ir) equally contributed as corresponding authors.

## Conflict of interest

The authors declare that they have no conflict of interest.

## Figures and Tables

**Table 1:**
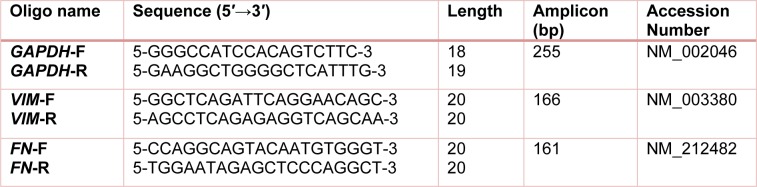
Oligonucleotide sequences and amplification fragment size

**Table 2:**
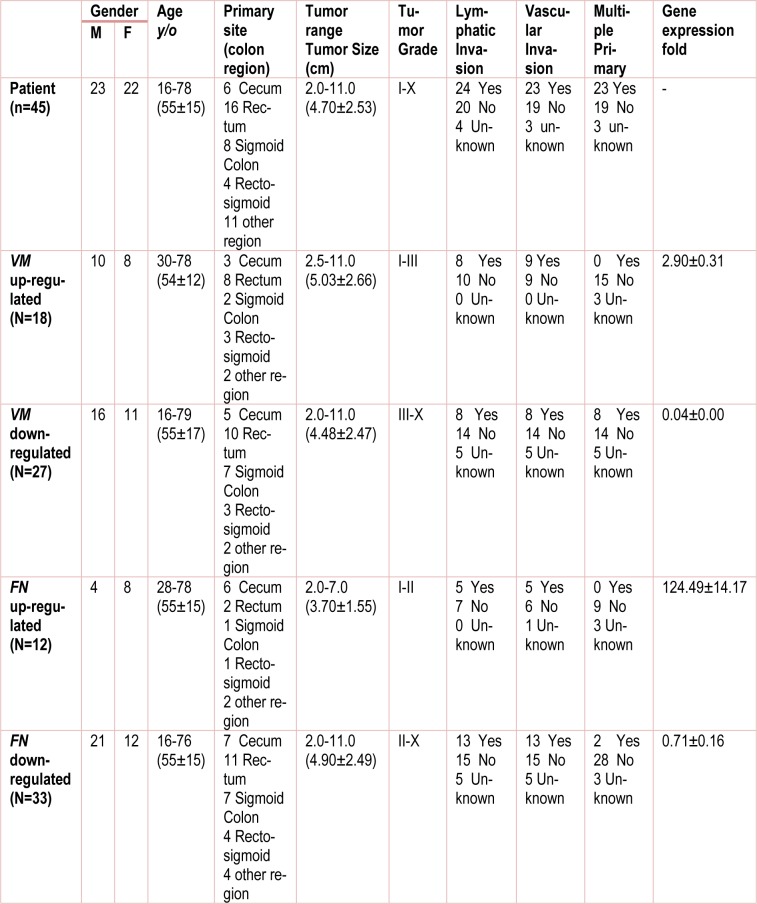
Demographic table including patient's age, gender, tumor size and tumor stage

**Table 3:**
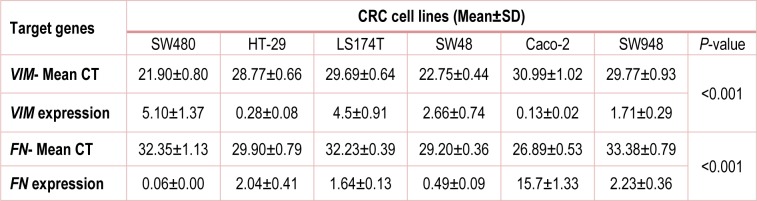
*VIM* and *FN* expression profile in CRC cell lines (Median±IQR)
